# Supplementary material for: Highly efficient light-emitting diodes via self-assembled InP quantum dots
Source: Nat Commun. 2025 May 7;16:4257. doi: 10.1038/s41467-025-59527-2 (PMC12059007; doi:10.1038/s41467-025-59527-2)
Supplement: Supplementary file 1 — Supplementary Information [file 41467_2025_59527_MOESM1_ESM.pdf]

Supplementary Information

Highly Efficient Light-Emitting Diodes via Self-Assembled InP Quantum Dots

Hui Li[1,2†], Jingyuan Zhang[3†], Wen Wen[2*], Yuyan Zhao[2], Hanfei Gao[2], Bingqiang Ji[4*], Yunjun Wang[5], Lei Jiang[1,2], Yuchen Wu[1,6*]

[1]Key Laboratory of Bio-inspired Materials and Interfacial Science, Technical Institute of Physics and Chemistry, Chinese Academy of Sciences, Beijing 100190, P. R. China.

[2]State Key Laboratory of Bioinspired Interfacial Materials Science, Suzhou Institute for Advanced Research, University of Science and Technology of China, Suzhou, Jiangsu 215123, P. R. China.

[3]Department of Physics, Shanxi Datong University, Datong 037009, P.R. China.

[4]School of Astronautics, Beihang University, Beijing 100191, China.

[5]Suzhou Xingshuo Nanotech Company, Ltd. (Mesolight), Suzhou 215123, China.

[6]University of Chinese Academy of Sciences (UCAS) Beijing 100049, P. R. China.

†These authors contributed equally to this work.

*Corresponding author. Emails: wen24@ustc.edu.cn (W.W.); bingqiangji@buaa.edu.cn (J.B.); wuyuchen@iccas.ac.cn (Y.W.)

Supplementary Figures

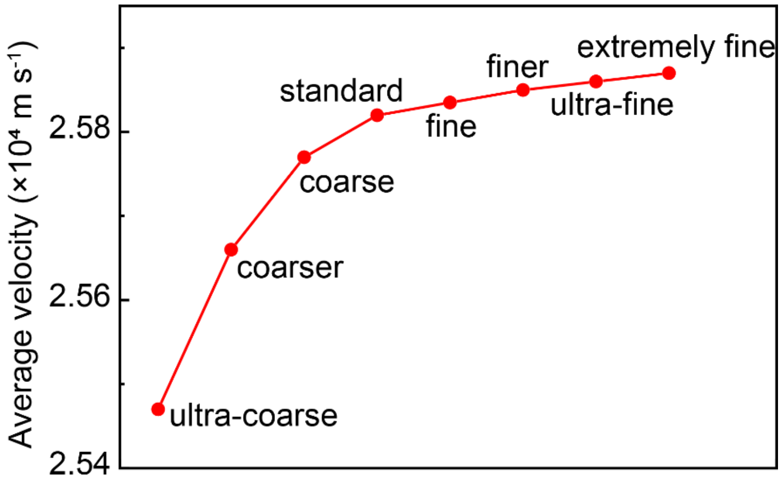

Supplementary Fig. 1 | Average velocity of the fluid field simulated under different mesh types.

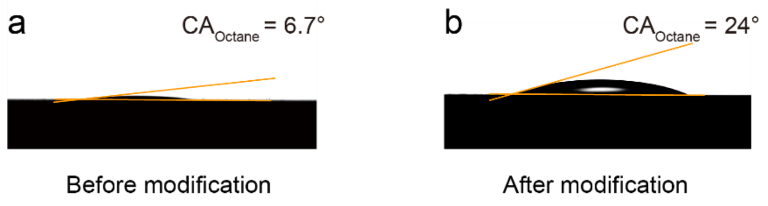

Supplementary Fig. 2 | Changes in CA before and after interfacial anti-adhesion modification using DMS.

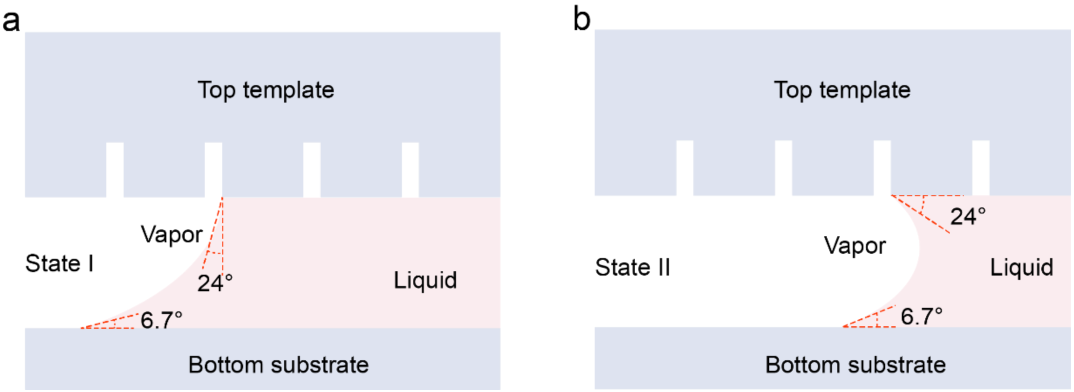

Supplementary Fig. 3 | Schematic diagram of the dewetting process of the solution.

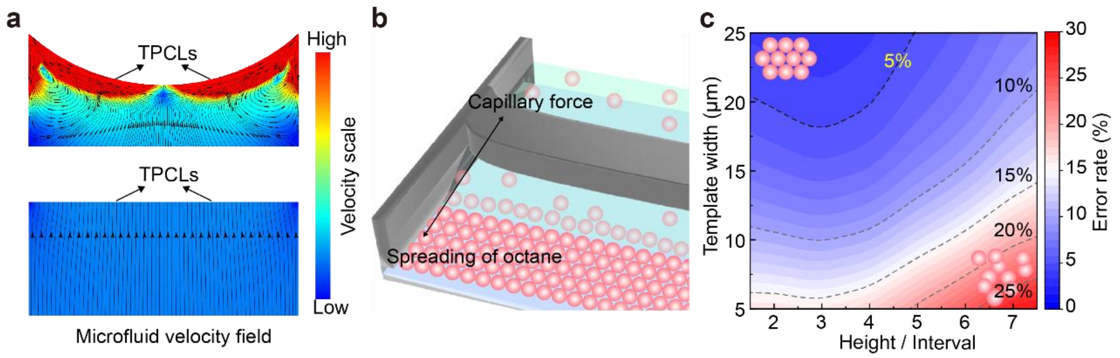

Supplementary Fig. 4 | Microscale fluid dynamics and capillary interactions in template-assisted QD film formation. a, Microfluid velocity field simulations of two liquid films: one with a micropillar template and the other without. The lines with arrows indicate the direction of fluid flow, while the color gradient from blue to red represents the fluid velocity, with blue indicating slower flow and red indicating faster flow. The presence of the micropillar template stabilizes the TPCL, transitioning it from a curved line to a straight one, and promotes the formation of a uniform, dense QD film by eliminating complex fluid dynamics and enabling more uniform mass transfer. **b**, Capillary force generated by the reverse-bent micropillars balances the spreading of octane. **c**, Simulation of the jumping process of the liquid film in the micropillar gap, depicting contour plots of the fault tolerance rate of templates with different widths at different liquid film height/gap ratios.

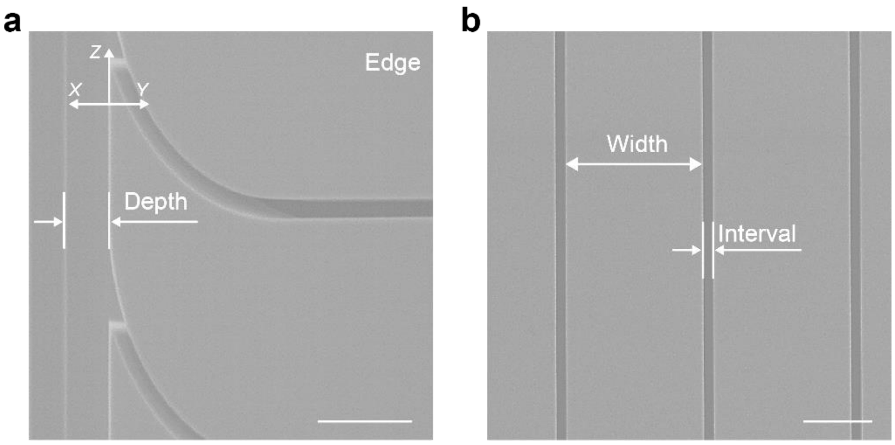

Supplementary Fig. 5 | SEM image of a micropillars template. The width of the micropillar template is 20 μm, an interval is 1μm, and a 3 μm depth. The edge has a reverse bending shape. Scale bar, 5 μm (a); 10 μm (b).

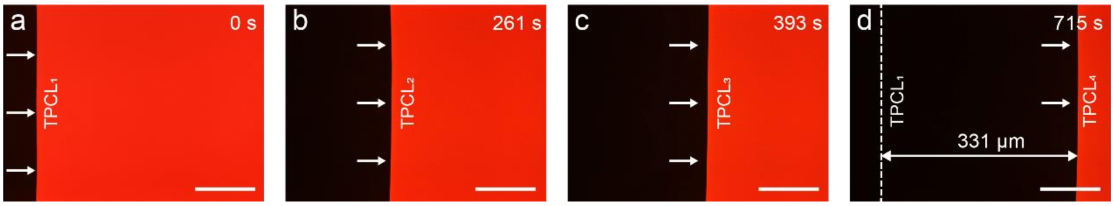

Supplementary Fig. 6 | In situ fluorescence microscopy image showing the retraction of the TPCL. All scale bar, 100 μm.

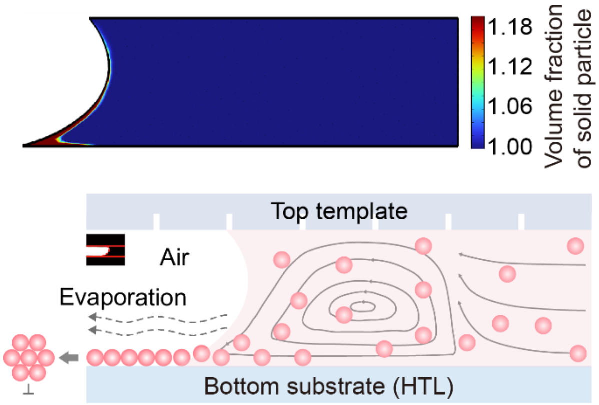

Supplementary Fig. 7 | Simulation of the volume fraction of solid particles and thermo-Marangoni flow in the liquid film.

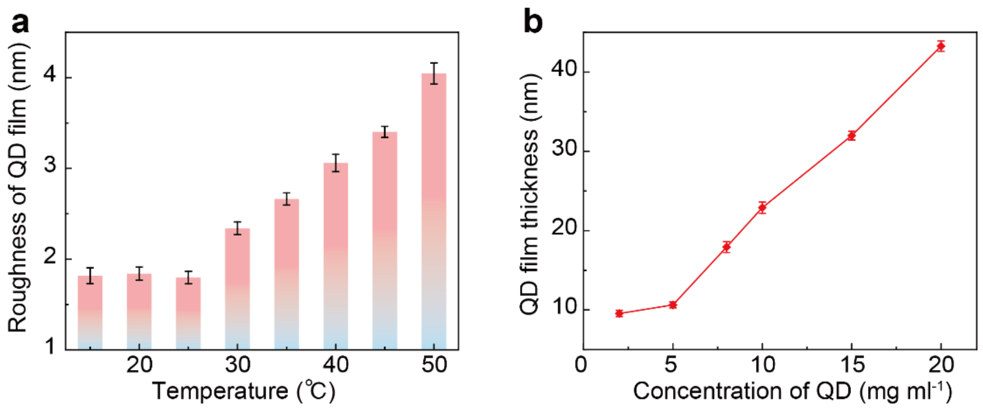

Supplementary Fig. 8 | Effect of temperature and concentration on QD film morphology. **a**, Relationship curve between ambient temperature and roughness of QD films. At 25°C and below, the surface roughness of QD film is low, and the surface roughness increases significantly with the increase of temperature. **b**, The relationship between the thickness of the QD film and the concentration of QD solution. The film thickness increases almost linearly with the concentration of QD solution. Error bars represent the standard deviation from five independent samples.

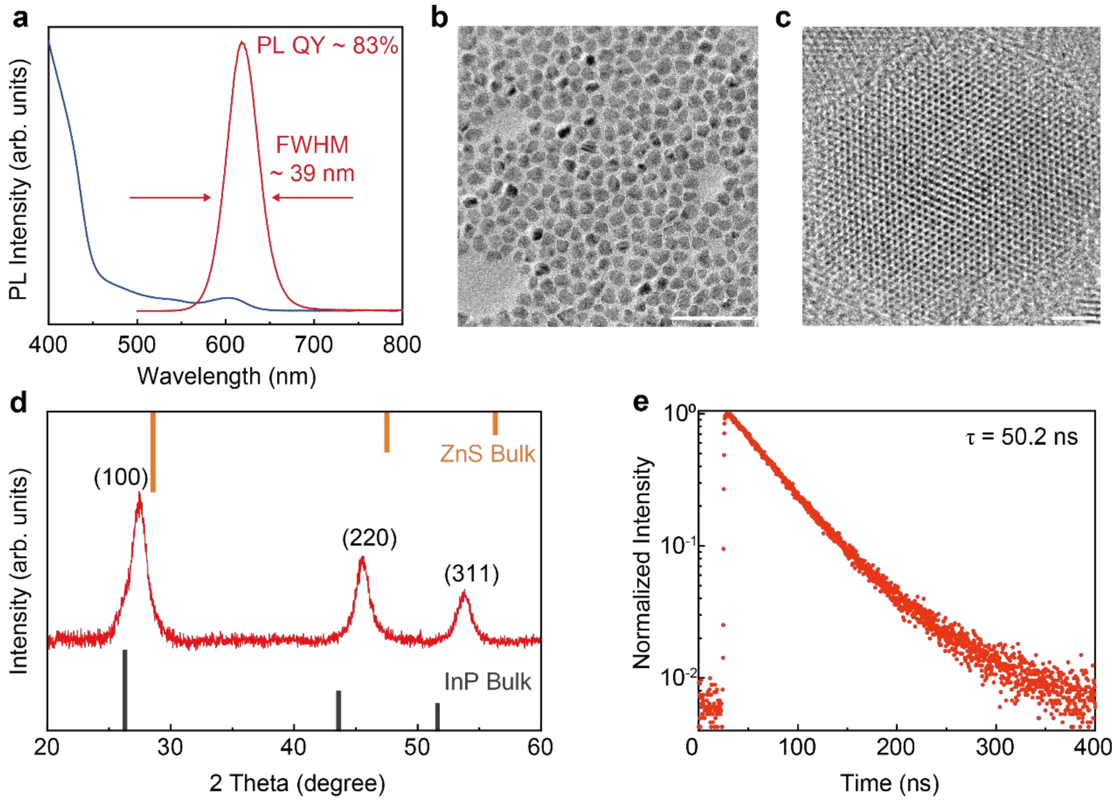

Supplementary Fig. 9 | Characterization of InP QDs. a, Fluorescence spectrum and ultraviolet absorption spectrum of InP QDs. The emission peak of the QDs is located at 622 nm, with PL QY of 83% and a FWHM of 39 nm. **b-c,** TEM and HRTEM images of InP QDs, confirming uniform morphology, good dispersion, and high crystallinity. **d,** XRD pattern of InP QDs, revealing the zinc blende structure of QDs. **e,** Photoluminescence decay curves of InP QDs in solution, exhibiting approximately single-exponential decay, indicating effective suppression of Auger recombination processes. Scale bar, 50 nm (b); 1 nm (c).

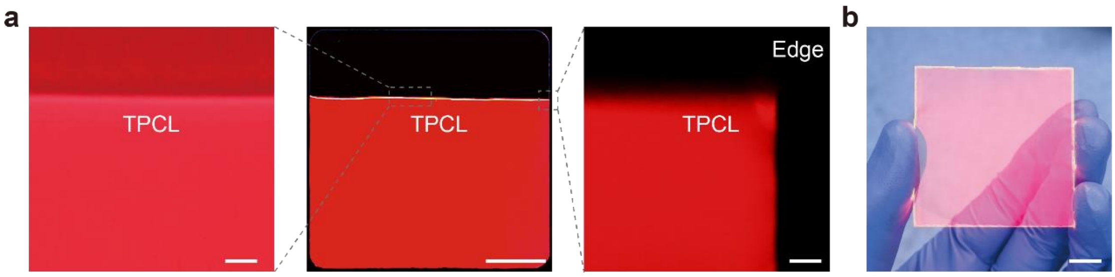

Supplementary Fig. 10 | Morphology of the TPCL during the dewetting process. a, During the dewetting process, both the central and edge TPCL remain a straight line. **b,** Large-area (5 × 5 cm^2) InP QD film. Scale bar, 10 µm, 500 µm, 10 µm (a); 1 cm (b).

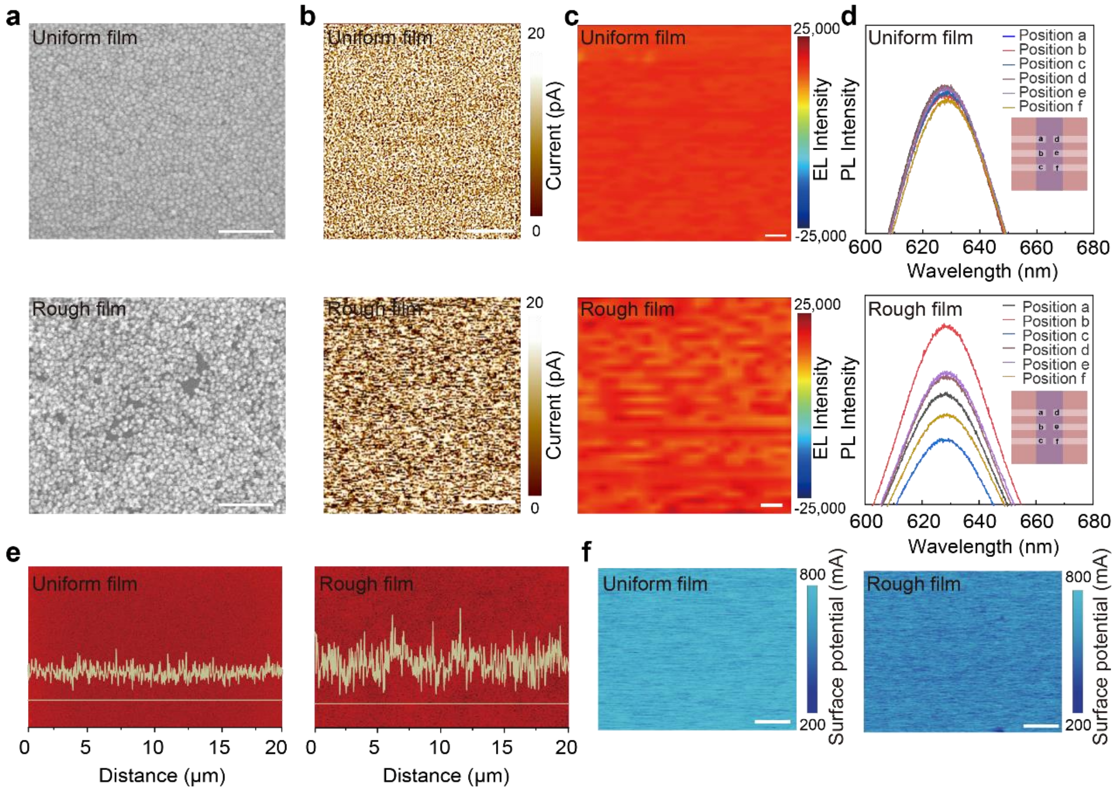

Supplementary Fig. 11 | Characterization of InP QD Films. a, SEM images of two types of QD films. Scale bar, 100 nm. **b,** Characterization of the electrical conductivity of two types of QD films. Scale bar, 1 μm. **c,** PL emission intensity maps of two types of QD films. Scale bar, 10 μm. **d,** PL emission intensity maps of two types of QD films in different operating regions. **e,** Confocal images and PL emission intensity maps of two types of QD films. Scale bar, 1 μm. **f,** Interface potential maps of two types of QD films. Scale bar, 1 μm.

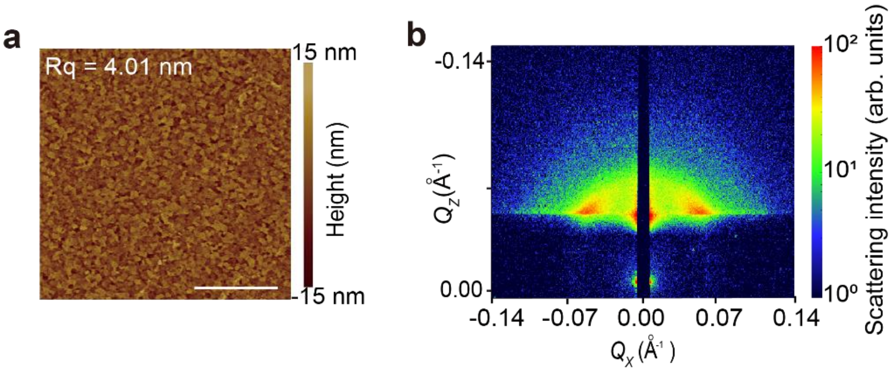

Supplementary Fig. 12 | Characterization of InP QD films prepared by traditional methods. a, Surface flatness diagram of InP QD film prepared by traditional method. Scale bar, 1 μm. **b,** GISAXS diagram of InP QD films prepared by traditional methods.

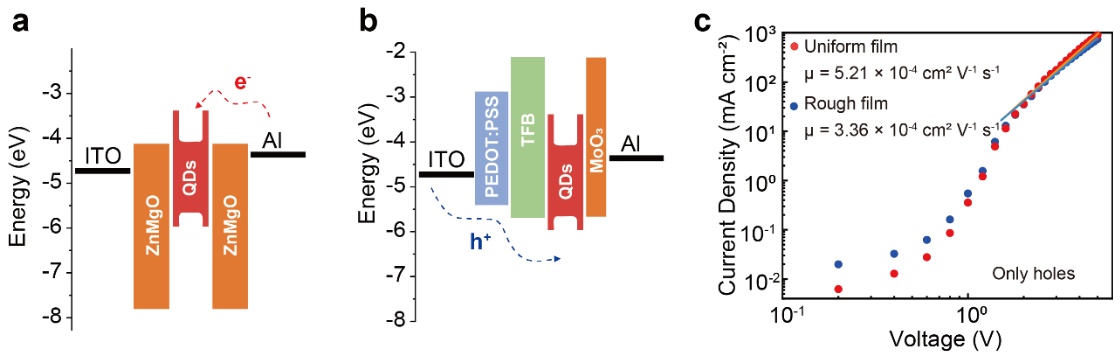

Supplementary Fig. 13 | Single carrier mobility obtained within the SCLC model. a, The electron only device consist of ITO/ZnMgO/QDs/ZnMgO/Al layers, the holes are blocked by the ZnMgO layer. **b**, The hole only device consist of ITO/PEDOT:PSS/TFB/QDs/MoO_3/Al layers, the electrons are blocked by the MoO_3 layer. **c**, The hole mobility of homogeneous and dense QD films and coarse QD films.

The electron only devices based on the evaporation-driven self-assembly strategy (device structure as shown in Supplementary Fig. 8a) exhibit a lower current density (*J*), while the hole only devices (device structure as shown in Supplementary Fig. 8c) have a slightly higher current density. The relationship between the *J* and voltage (*V*) for both types of electron only and hole only devices follows the characteristics of space charge limited current (Supplementary Fig. 8b and Supplementary Fig. 8d). By fitting the *J*-*V* curves using the Mott-Gurney law, the charge mobility (μ can be calculated by): [1]

$$J = \frac{9}{8}\varepsilon_0\varepsilon_r\mu\frac{V^2}{L^3} \tag{1}$$

where ε_0 is the vacuum permittivity, $\varepsilon_r = 12.1$ is the relative permittivity of QDs[2], and L is the channel length. Higher electron (hole) mobility of 6.14×10^{-4} cm^2 V^{-1} s^{-1} (5.21×10^{-4} cm^2 V^{-1} s^{-1}) can be observed on evaporation-driven self-assembled QD films, while the spin-coated film exhibits a lower mobility of 4.39×10^{-4} cm^2 V^{-1} s^{-1} (3.36×10^{-4} cm^2 V^{-1} s^{-1}).

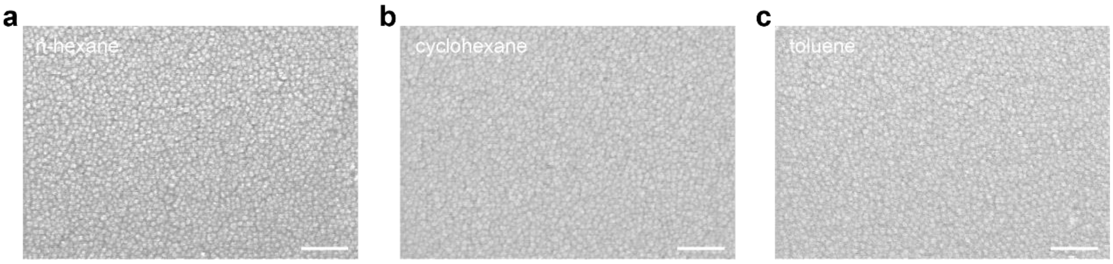

Supplementary Fig. 14 | Comparison of QD films prepared with different solvents. The image shows the films prepared using n-hexane, cyclohexane, and toluene alongside those prepared with n-octane. The results reveal that QD films from all solvents exhibit comparable uniformity and density, demonstrating the versatility of the evaporation-driven self-assembly strategy across different solvent systems. All scale bar, 100 nm.

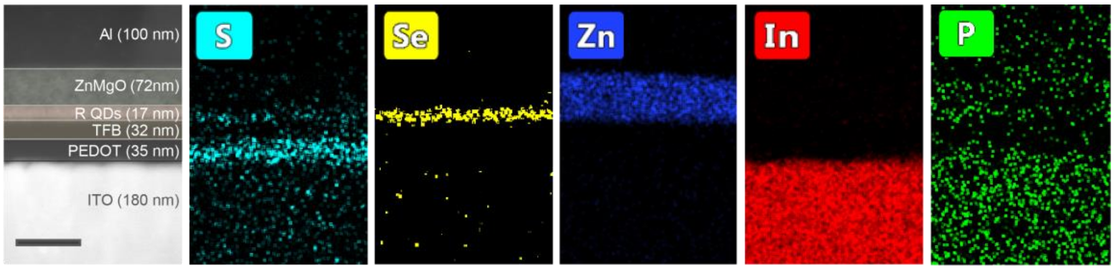

Supplementary Fig. 15 | HAADF-STEM images of the cross-section of an InP-based QLED. Energy-dispersive X-ray spectroscopy (EDX) elemental maps of S, Se, Zn, In, and P within the QLED structure clearly show the distribution of these elements, highlighting the thickness and position of each functional layer. Scale bar: 100 nm.

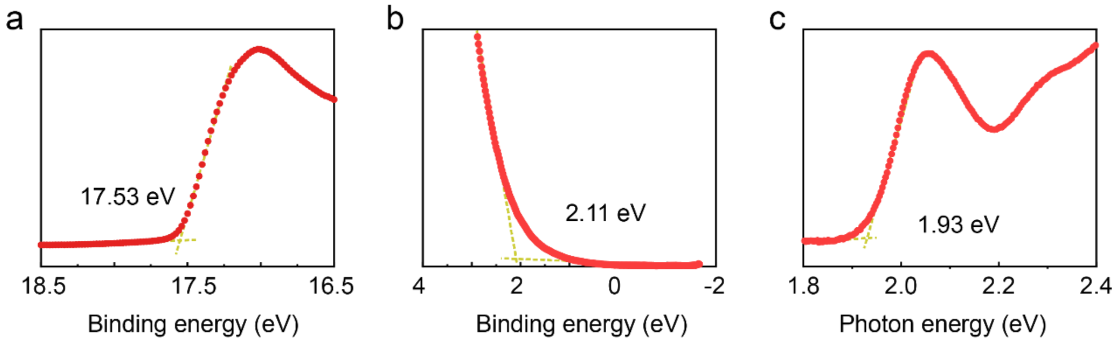

Supplementary Fig. 16 | UPS spectra and UV-Vis absorption spectra of InP QDs. a-b, Zoomed spectra of the secondary electron threshold and valence band (VB) region. The VB energy level of InP QDs is determined to be 5.80 eV. The bandgap of InP QDs, as determined by absorption spectroscopy, is 1.93 eV. Therefore, the CBs of regular and large QDs are determined to be 3.87 eV. **c,** UV-Vis absorption spectra of InP QDs.

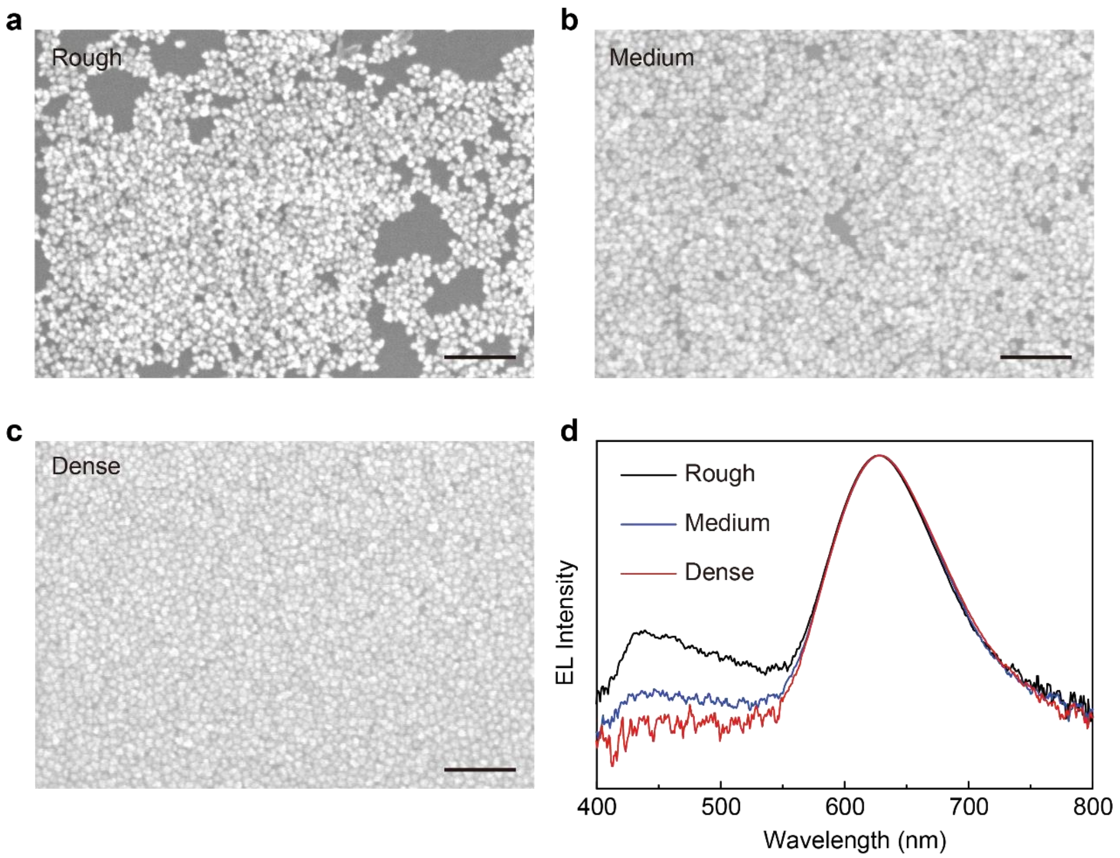

Supplementary Fig. 17 | SEM images of red InP-based QD films with varying uniformity and density and corresponding EL spectra of QLEDs. a, SEM image of a rough QD film. **b**, SEM image of a medium-density QD film. **c**, SEM image of a dense QD film. **d**, EL spectra of red InP-based QLEDs corresponding to films with different uniformities and densities. As the uniformity and density of the QD film increase, the emission intensity in the blue region gradually decreases and eventually disappears. All scale bar, 100 nm.

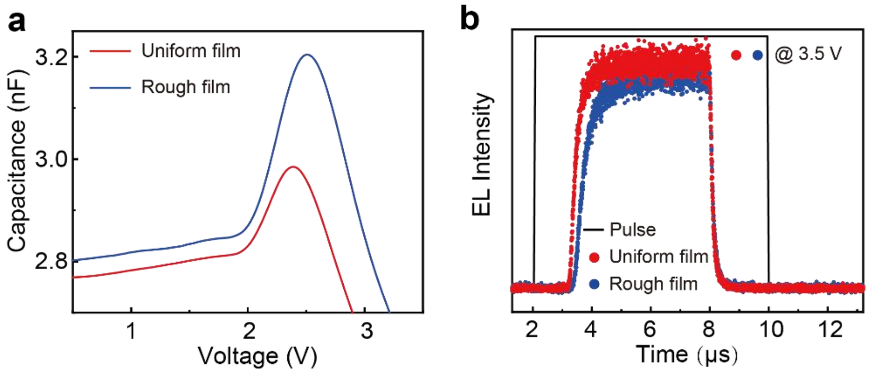

Supplementary Fig. 18 | Capacitance-voltage characteristics and transient electroluminescence (TrEL) measurements of QLED devices with two types of QD films. **a**, Capacitance-voltage characteristics. When the applied voltage exceeds 2 V, the capacitance of both devices increases, indicating charge carrier accumulation at the TFB/QD interface. As the voltage rises to 2.5 V, the capacitance begins to decrease, suggesting breakdown of the capacitance at the TFB/QD interface due to sufficient hole injection. The QLED with the uniform, dense QD film exhibits lower capacitance across the entire voltage range, indicating reduced charge accumulation. **b**, TtrEL results. At 3.5 V, the QLED with the uniform, dense QD film shows shorter turn-on times and faster transient electroluminescence. These results indicate that the uniform, dense QD film enhances the electron-hole radiative recombination rate while significantly suppressing charge leakage.

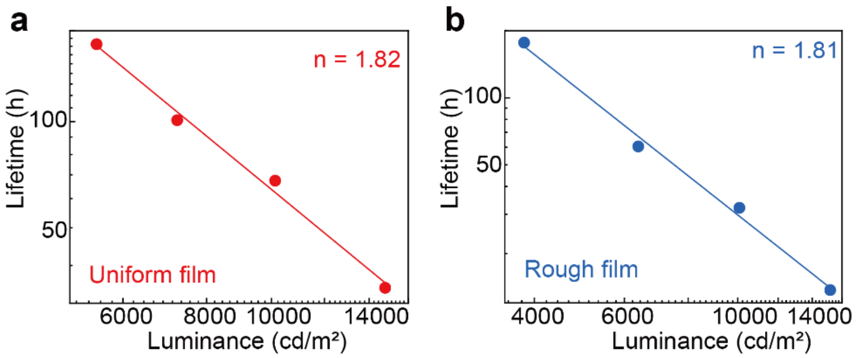

Supplementary Fig. 19 | T_{50} lifetime measurements of two InP-based QLEDs at different initial luminance.

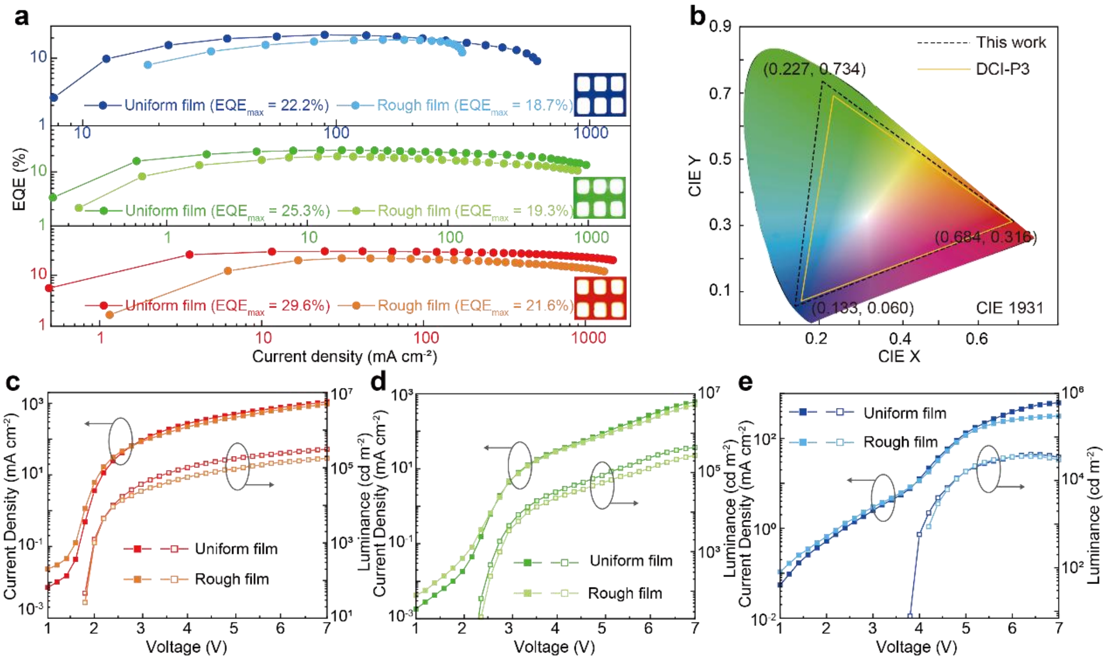

Supplementary Fig. 20 | Electrical performance for Cd-based QLEDs. a, Compared to the maximum EQEs of 21.6%, 19.3%, and 18.7% for red, green, and blue QLEDs based on rough QD films, the maximum EQEs for QLEDs based on dense QD films are significantly improved to 29.6%, 25.3%, and 22.2%, respectively. This is one of the best results reported so far. **b**, CIE 1932 color space coordinates corresponding to the red, green, and blue Cd-based QLEDs in (a), with specific CIE coordinates of (0.684, 0.316), (0.227, 0.734), and (0.133, 0.060). The yellow triangle represents the DCI-P3 color space. These color gamuts surpass the Digital Cinema Initiatives P3 (DCI-P3) color space, a standard for wide-gamut displays. **c-e**, Devices based on dense QD films exhibit lower current density in the ohmic contact region compared to those based on rough QD films, indicating effective suppression of leakage current. Additionally, devices with dense QD films show enhanced luminance: red Cd-based QLEDs (c) increased from 2.1 × 10^5 to 3.6 × 10^5 cd m^{-2}, green Cd-based QLEDs (d) from 3.8 × 10^5 to 5.6 × 10^5 cd m^{-2}, and blue Cd-based QLEDs (e) from 3.6 × 10^4 to 4.0 × 10^4 cd m^{-2}, compared to devices with rough QD films.

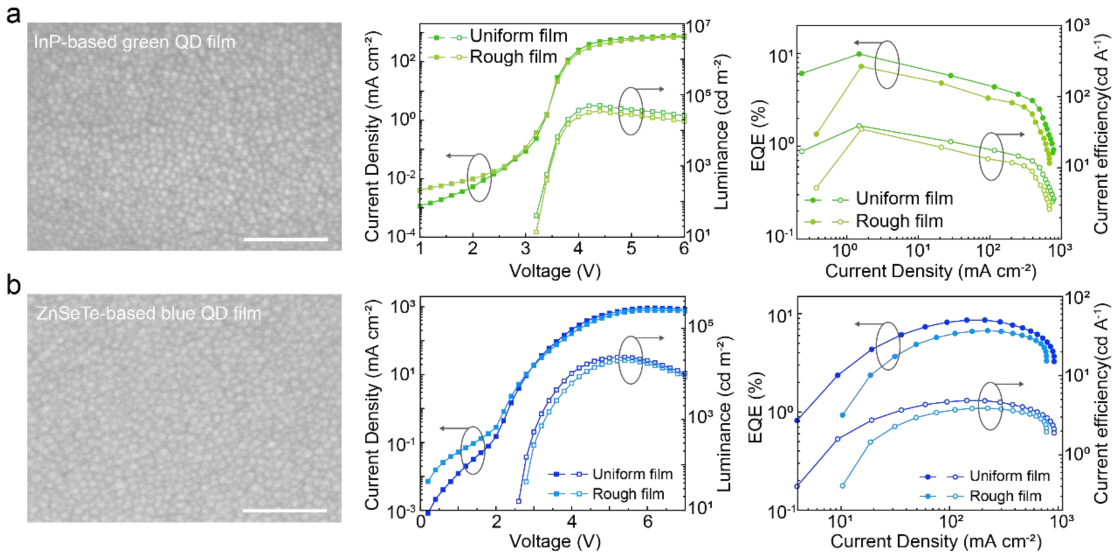

Supplementary Fig. 21 | Electrical performance for green InP-based and blue ZnSeTe-based QLEDs. a, SEM images of green InP-based QD film prepared via the evaporation-driven self-assembly strategy. Scale bar, 100 nm. Current density-luminance-voltage and EQE-current efficiency-current density characteristics of green InP-based QLEDs. Compared to QLEDs fabricated using conventional methods, those with uniform QD films exhibit significantly lower current density in the ohmic contact region, with the EQE increasing from 7.2% to 9.8% and the luminance improving from 3.5×10^4 cd m^{-2} to 5.0×10^4 cd m^{-2}. **b**, SEM images of blue ZnSeTe-based QD film prepared via the evaporation-driven self-assembly strategy. Scale bar, 100 nm. Current density-luminance-voltage and EQE-current efficiency-current density characteristics of green InP-based QLEDs. Compared to QLEDs fabricated using conventional methods, those with uniform QD films exhibit significantly lower current density in the ohmic contact region, with the EQE increasing from 7.2% to 9.8% and the luminance improving from 3.5×10^4 cd m^{-2} to 5.0×10^4 cd m^{-2}.

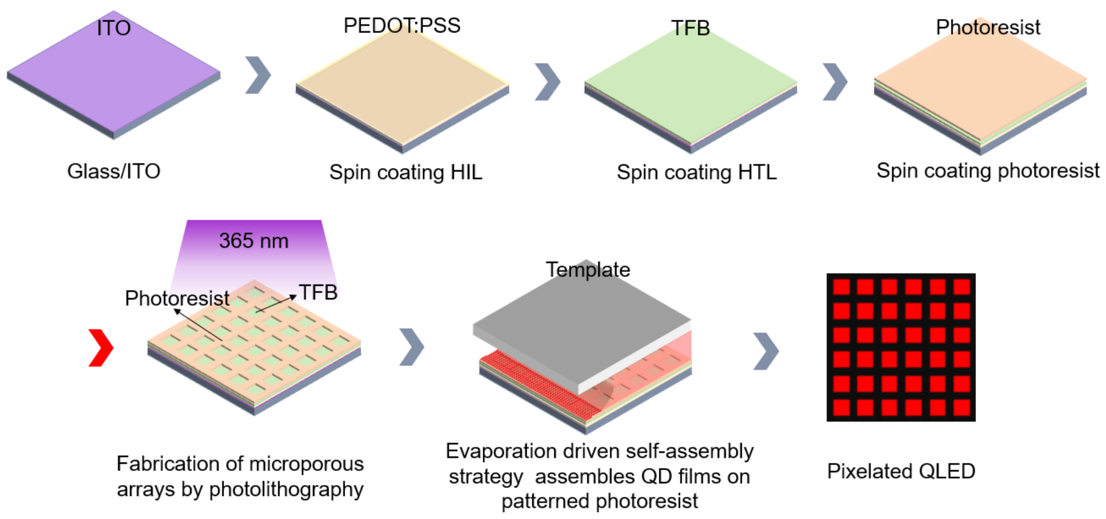

Supplementary Fig. 22 | Schematic diagram of preparation process of patterned QLED.

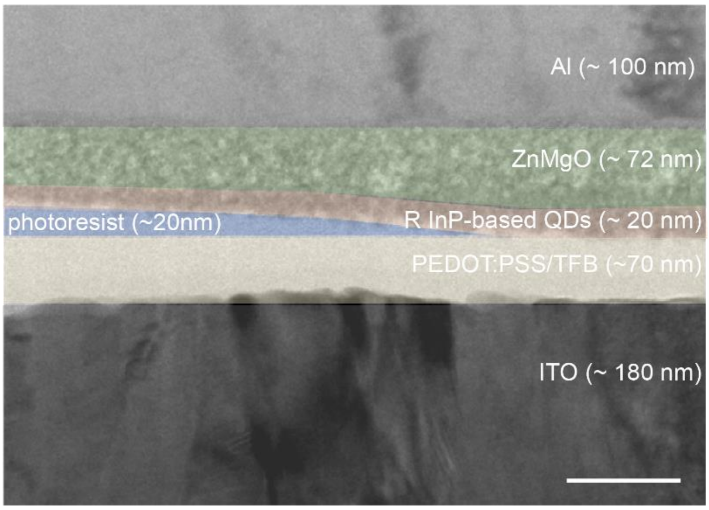

Supplementary Fig. 23 | Cross-sectional TEM image of patterned QLED. Scale bar, 100 nm.

Supplementary Tables

Supplementary Table 1. **Average velocity of the fluid field under different meshes.**

**Serial number**	**Mesh refinement level.**	**Average velocity（$\times 10^4$ m s^{-1}）**
1	ultra-coarse mesh	2.547
2	coarser mesh	2.566
3	coarse mesh	2.577
4	standard mesh	2.582
5	fine mesh	2.5835
6	finer mesh	2.585
7	ultra-fine mesh	2.586
8	extremely fine mesh	2.587

Supplementary Table 2. **Performance comparison of the devices developed in this work with other high-performance red InP-based QLEDs.**

Year	PL (nm)	EQE_{max} (%)	L_{max} (cd m^{-2})	Ref.
2018	607	6.6	1,700	3
2019	618	12.2	10,000	4
2019	630	21.4	100,000	5
2020	632	21.8	23,300	6
2021	630	18.6	128,577	7
2021	-	18.3	>10,000	8
2022	620	22.2	110,000	9
2022	625	16.8	136,090	10
	621	22.5	107,160	
**2025**	**622**	**26.6**	**140,000**	**This work**

Supplementary Notes

Supplementary Note 1: Conductivity measurement

We used the substrate/QD architecture as a field-effect transistor to determine the film conductivity (σ) (as shown in Fig. 2f). Both films exhibited linear current-voltage (*I–V*) characteristics within the scanning voltage range of 0 V to 4 V. The σ of each film was extracted by plotting the current as a function of the scanning voltage:

$$\sigma = \frac{I}{V} \times \frac{L}{T \times W} \tag{2}$$

Where L is the channel length, set to 2 μm, T is the film thickness (~20 nm), W is the channel width, set to 100 μm, and I/V is the slope of the current-voltage curve. The calculated σ of the QD film prepared via the self-assembly strategy is 6.6×10^{-4} S m^{-1}, which is 2.75 times higher than that of the QD film prepared via the spin-coating method (σ =2.4×10^{-4} S m^{-1}).

Supplementary Note 2: Particle tracking simulation explanation

We utilized the Particle Tracing Module within COMSOL Multiphysics 6.2 to simulate the motion of particles in space. In the simulation, particles are introduced into the fluid as point masses at the inlet. The forces acting on the particles, including fluid drag, gravity, and Brownian motion, are simultaneously considered to analyze the particle behavior and determine their movement trends within the diffusive medium. All the formulas in Supplementary Note 3 are sourced from the software manual.

The relationship between the fluid particle tracking motion and the acting forces on the particles is given by the following equation:

$$\frac{d}{dt}(m_p V) = F_D + F_g + F_{ext} \tag{3}$$

$$V = \frac{dq}{dt} \tag{4}$$

where m_p is the particle mass, V is the particle velocity vector, q is the particle position variable, F_D and F_g represent the fluid drag force and gravitational force, respectively, and F_{ext} is the external force, which is zero in this case.

The fluid drag force is expressed as:

$$F_D = \left(\frac{1}{\tau_p}\right) m_p (U - V) \tag{5}$$

where τ_p is the particle velocity response time and U is the fluid velocity vector at the particle's location.

The particle velocity response time τ_p quantifies the timescale for the particle's

velocity to equilibrate with the surrounding fluid velocity. According to the Stokes drag law, τ_p is given by:

$$\tau_p = \frac{\rho_p d_p^2}{18\mu} \quad (6)$$

where ρ_p is the particle density, d_p is the particle diameter and μ is the dynamic viscosity of the fluid.

The gravitational force term includes buoyancy, taking into account the density of the surrounding fluid. Therefore, the gravitational force F_g is expressed as:

$$F_g = m_p g \frac{(\rho_p - \rho)}{\rho_p} \quad (7)$$

where ρ is the density of the surrounding fluid.

To account for the random motion of particles at the microscopic scale, the Brownian motion force is included, as represented by the equation:

$$F_b = \zeta \sqrt{\frac{12\pi k_B \mu T r_p}{\triangle t}} \quad (8)$$

Where $\triangle t$ is the solver time step, r_p is the particle radius, T is the fluid temperature, k_B is the Boltzmann constant and ζ is a normally distributed random number.

Supplementary Note 3: Mesh independence test

To ensure the reliability of the numerical simulation results, we performed a mesh independence test to verify whether the simulation results stabilize with mesh refinement, thus eliminating the influence of mesh discretization on the computational outcomes. In this test, we compared eight different mesh densities: ultra-coarse mesh, coarser mesh, coarse mesh, standard mesh, fine mesh, finer mesh, ultra-fine mesh, and extremely fine mesh. The differences between these mesh types mainly lie in the number of elements and the mesh size. The ultra-coarse mesh has the largest mesh size and the fewest elements, while the extremely fine mesh has the smallest mesh size and the most elements. For each mesh type, simulations were run under the same conditions, and the average velocity of the fluid field for each mesh was compared (Supplementary Table 1). The test results indicate that as the mesh is refined from the standard mesh to the finer meshes, the average velocity of the fluid field gradually stabilizes (Supplementary Fig. 1). Therefore, the refined mesh can be considered numerically convergent. Based on this, we selected any mesh from the fine to extremely fine range for further simulation analysis to ensure the accuracy and reliability of the results.

Supplementary References

1. Shen, H. et al. Visible quantum dot light-emitting diodes with simultaneous high brightness and efficiency. *Nat. Photon.* **13**, 192–197 (2019).
2. Li. S. et al. Charge Transport in Blue Quantum Dot Light-Emitting Diodes. *Adv. Electron. Mater.* 2400142 (2024).
3. Cao, F. et al. A layer-by-layer growth strategy for large-size InP/ZnSe/ZnS core–shell quantum dots enabling high-efficiency light-emitting diodes. *Chem. Mater.* **30**, 8002-8007 (2018).
4. Li, Y. et al. Stoichiometry-controlled InP-based quantum dots: synthesis, photoluminescence, and electroluminescence. *J. Am. Chem. Soc.* **141**, 6448-6452 (2019).
5. Won, Y. H. et al. Highly efficient and stable InP/ZnSe/ZnS quantum dot light-emitting diodes. *Nature* **575**, 634-638 (2019).
6. Yeom, J. E. et al. Good charge balanced inverted red InP/ZnSe/ZnS-quantum dot light-emitting diode with new high mobility and deep HOMO level hole transport layer. *ACS Energy Lett.* **5**, 3868-3875 (2020).
7. Han, M. G. et al. InP-based quantum dot light-emitting diode with a blended emissive layer. *ACS Energy Lett.* **6**, 1577-1585 (2021).
8. Jeong, B. G. et al. Interface polarization in heterovalent core-shell nanocrystals. *Nat. Mater.* **21**, 246-252 (2022).
9. Li H. et al. ZnF_2-assisted synthesis of highly luminescent InP/ZnSe/ZnS quantum dots for efficient and stable electroluminescence. *Nano Lett.* **22**, 4067-4073 (2022).
10. Li H. et al. High performance InP-based quantum dot light-emitting diodes via the suppression of field-enhanced electron delocalization. *Adv. Funct. Mater.* **32**, 2204529 (2022).
