# Supplementary material for: Highly efficient light-emitting diodes via self-assembled InP quantum dots
Source: Nat Commun. 2025 May 7;16:4257. doi: 10.1038/s41467-025-59527-2 (PMC12059007; doi:10.1038/s41467-025-59527-2)
Supplement: Supplementary file 2 — Description of Additional Supplementary Files [file 41467_2025_59527_MOESM2_ESM.pdf]

Description of Additional Supplementary Files

Supplementary Movie 1. QD solution slides off the PDMS-modified substrate, leaving no noticeable residue along the trajectory.
